# Identification of *cis*-regulatory sequences reveals potential participation of lola and Deaf1 transcription factors in *Anopheles gambiae* innate immune response

**DOI:** 10.1371/journal.pone.0186435

**Published:** 2017-10-13

**Authors:** Bernardo Pérez-Zamorano, Sandra Rosas-Madrigal, Oscar Arturo Migueles Lozano, Manuel Castillo Méndez, Verónica Valverde-Garduño

**Affiliations:** 1 Departamento de Infección e Inmunidad, Centro de Investigaciones Sobre Enfermedades Infecciosas, Instituto Nacional de Salud Pública, Cuernavaca, Morelos, México; 2 Escuela de Salud Pública de México, Instituto Nacional de Salud Pública, Cuernavaca, Morelos, México; 3 Winter Genomics, Lindavista, Ciudad de México, México; Mayo Clinic Arizona, UNITED STATES

## Abstract

The innate immune response of *Anopheles gambiae* involves the transcriptional upregulation of effector genes. Therefore, the cis-regulatory sequences and their cognate binding factors play essential roles in the mosquito’s immune response. However, the genetic control of the mosquito’s innate immune response is not yet fully understood. To gain further insight on the elements, the factors and the potential mechanisms involved, an open chromatin profiling was carried out on *A*. *gambiae*-derived immune-responsive cells. Here, we report the identification of *cis*-regulatory sites, immunity-related transcription factor binding sites, and *cis*-regulatory modules. A *de novo* motif discovery carried out on this set of *cis*-regulatory sequences identified immunity-related motifs and *cis*-regulatory modules. These modules contain motifs that are similar to binding sites for REL-, STAT-, lola- and Deaf1-type transcription factors. Sequence motifs similar to the binding sites for GAGA were found within a *cis*-regulatory module, together with immunity-related transcription factor binding sites. The presence of Deaf1- and lola-type binding sites, along with REL- and STAT-type binding sites, suggests that the immunity function of these two factors could have been conserved both in *Drosophila* and *Anopheles gambiae*.

## Introduction

Some mosquito species are vectors of infectious microbes that cause human disease. *Anopheles gambiae* is the main vector of *Plasmodium* parasites in sub-Saharan Africa. Despite the application of multiple strategies in order to control malaria transmission, these parasites still kill hundreds of thousands of people, mainly children, around the world. It has been recently demonstrated that, during the sporogonic cycle, the passage of the parasite through the mosquito tissues results in a radical modification of its virulence [1]. This finding underscores the relevance continuing to carry out more intense and detailed studies on the interactions between the mosquitoes and the invading microorganisms. An important component of these interactions is the vigorous innate immune response of mosquitoes when their tissues are invaded by a diversity of microorganisms. The innate immune response of *A*. *gambiae* relies largely on the transcriptional activation of the effector genes. This has been confirmed by multiple studies on the transcriptional profiling of the innate immune response of mosquitoes [2–4]. These transcriptomic studies have revealed the differential transcription of some immunity effector genes, both *in vivo* and *in vitro* (4a-3B cells). In the immune-responsive cell, the transcripts of a small number of immunity genes are increased by a diversity of immunity challenges. However, not only the genes identified as immunity genes become upregulated; this points to the presence of additional physiological changes upon immune stimulation. The transcription of each gene can be regulated by a number of *cis*-regulatory sequences, which can be proximal, such as promoters, or distal, such as enhancers. Distal *cis*-regulatory sequences can be located hundreds of kilo bases (Kb) away from their target gene transcriptional start site (TSS). These regulatory sequences contribute to establish expression levels through DNA-protein interactions involving transcription factors (TFs) and other regulatory factors. Key TFs involved in the transcriptional regulation of the innate immune response of mosquitoes have been identified. These include immunity-related REL [5] and C/EBPalpha TFs, both of which have been shown to be involved in the regulation of the *Defensin 1* gene (*Def1*) [6, 7]. Two STAT transcription factors participating in the transcription regulation of the immune response of *A*. *gambiae* have also been identified [8]. Also a LITAF-like factor (LL3) involved in the clearance of *Plasmodium* parasites in the midgut epithelium of *A*. *gambiae* [9]. However, the current understanding of the genetic control of the innate immune response of mosquitoes is still incomplete. Despite the identification of key immunity TFs, no specific mechanisms of genetic control have been described in *A*. *gambiae*.

Additional factors and mechanisms, which have not yet been identified, may play important roles in defining the transcriptional profiles generated by an immune challenge in *A*. *gambiae*. An important step towards uncovering these mechanisms and factors is the identification of *cis*-regulatory sequences. Therefore, we set out to identify the *cis*-regulatory sequences, the transcription factor binding sites (TFBSs), the *cis*-regulatory modules (CRMs), and the candidate factors potentially involved in the innate immune response of these mosquitoes.

Hemocytes play key roles in the cellular and humoral innate immune response of the mosquitoes. However, there are only about two to four thousand hemocytes in each mosquito [10]. Only a small fraction of the total DNA (under 5%) is involved in the *cis*-regulatory function in a given cell type in metazoans. Therefore, we used an *A*. *gambiae* 4a-3B hemocyte-like cell line to obtain enough *cis*-regulatory DNA. These cells have been previously used to characterize the immune response of the mosquitoes. Many immunity genes activated in mosquitoes have also been found to be upregulated when these cells are stimulated during the immune response [4, 11]. This indicates that this cell line is well suited to identify the *cis*-regulatory sequences involved in the innate immune response of *A*. *gambiae*. Open chromatin is a hallmark of the *cis*-regulatory function and, in contrast to ChIP-seq, it does not require specific antibodies [12–14]. TFBSs within open chromatin sites have been shown to be robust candidates for *in vivo* occupancy [15]. Here, we report the genome-wide identification of *cis*-regulatory sequences in a hemocyte-like immune-responsive *A*. *gambiae*-derived cell line. A *de novo* motif discovery applied to these *cis*-regulatory sequences shows significant enrichment of motifs, similar to the binding sites of immune transcription factors. These motifs frequently co-occur within *cis*-regulatory sequences to form immunity-related *cis*-regulatory modules (CRMs). Among these immunity-related sequence motifs, we found that some are similar to the binding sites for *Drosophila’s* lola- and Deaf1-type TFs. These motifs are also within CRMs, together with other motifs matching *A*. *gambiae*’s innate immunity TFBSs, such as those for REL- and STAT-type TFs. These data suggests potential conservation of the role of Deaf1- and lola-type TFs in innate immunity both in *A*. *gambiae* and *Drosophila*. We provide genomic coordinates for the set of open chromatin sites identified in this work.

## Results

### Open chromatin profiling

**Fig 1:**
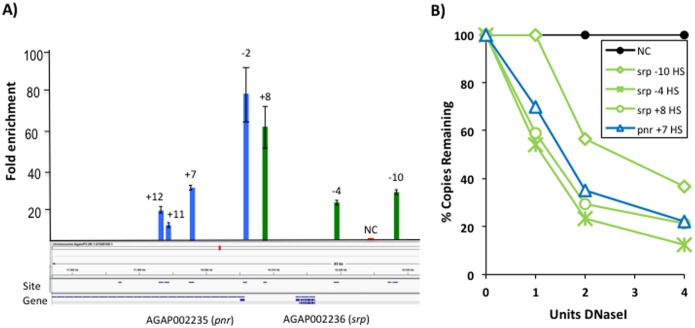
FAIRE-qPCR and DNaseI-qPCR validation of selected *cis*-regulatory sites detected by FAIRE-seq. (A) Cis-regulatory sites identified at the locus of *A*. *gambiae*’s putative orthologous gene to *Drosophila* hematopoietic *serpent* gene were confirmed by FAIRE-qPCR. The cis-regulatory sites identified by FAIRE-seq at -10 Kb, -4 Kb and +8 Kb from *A*. *gambiae*’s putative serpent gene (AGAP002236, ensembl!) are enriched in independently prepared open chromatin samples. The *cis*-regulatory sites identified by FAIRE-seq at -2 Kb, +7 Kb, +11 Kb and +12 Kb from *A*. *gambiae*’s putative pannier gene locus (AGAP002235) are also enriched in independently prepared open chromatin samples. (B) Nuclease sensitivity assay by DNaseI-qPCR for some open chromatin sites identified by FAIRE-seq, also detected by FAIRE-qPCR. The genomic coordinates for the tested cis-regulatory sites are relative to the annotated transcriptional start site of each gene.

In order to experimentally identify the active *cis*-regulatory sites in *A*. *gambiae*-derived 4a-3B immune competent cells, a genome-wide open chromatin profiling was carried out by FAIRE-seq. A total of 19 919 open chromatin sites were identified from open chromatin DNA libraries derived from 4a3B cells, as detected from the resulting sequencing reads through a MACS algorithm with a cut-off *p-*value threshold of 10^−8^. Since there are no previously determined *cis*-regulatory sequence datasets in this species, peak detection was also carried out by DFilter [16] to confirm the robustness of our data. Applying a cut-off *p-*value threshold of 1x10^-8^, this algorithm identified 38 305 open chromatin, *cis*-regulatory sites. The comparison of the sites identified by MACS and the sites identified by DFilter produced an overlapping set of 26 440 *open chromatin* sites (S1 File: **Dataset**), since a single site identified by MACS may contain a number of shorter sites identified by DFilter. Most sites (69%) were identified using both algorithms. Furthermore, the MAnorm analysis [17] indicated that more than 80% of the sites were present in both biological replicates (Figure A in S2 File). This indicates our data are robust, and they allow a high-sensitivity detection of open chromatin *cis*-regulatory sites. A *locus* was then selected to validate the individual *cis*-regulatory sites by a FAIRE-qPCR and DNase I-qPCR sensitivity assay. This *locus* is located on chromosome 2R, and it contains gene AGAP002236. According to the ensembl! database, this gene is a putative orthologous gene to *serpent*, a *Drosophila* gene coding for a hematopoietic GATA-type TF [18]. This TF has been shown to participate in fly hematopoiesis, and it is, therefore, expected that there will be active *cis*-regulatory sites driving the expression of this orthologous gene in the hemocyte-like *A*. *gambiae*-derived cell line. This *locus* also contains gene AGAP002235, a putative orthologous gene to *Drosophila*’s *pannier*, according to OrthoDB [19], as well as other putative members of the GATA-type family of TFs. Fig 1A shows that the sites detected by FAIRE-seq within this *locus* can also be detected by a FAIRE-qPCR assay in independently prepared samples. In addition, some of these individually validated sites were also verified using an independent nuclease-sensitivity assay (Fig 1B). Although it is known that not all sites detected using a FAIRE assay can be detected using a DNaseI assay (nor *vice versa*), four of the FAIRE-tested sites were shown to be hypersensitive to DNaseI, confirming their status as open chromatin, *cis*-regulatory sites. These data on individually confirmed sites are consistent with the robust detection of *cis*-regulatory sequences using the genome-wide open chromatin profiling approach applied in this study.

### Genomic distribution of open chromatin *cis*-regulatory sites

**Fig 2:**
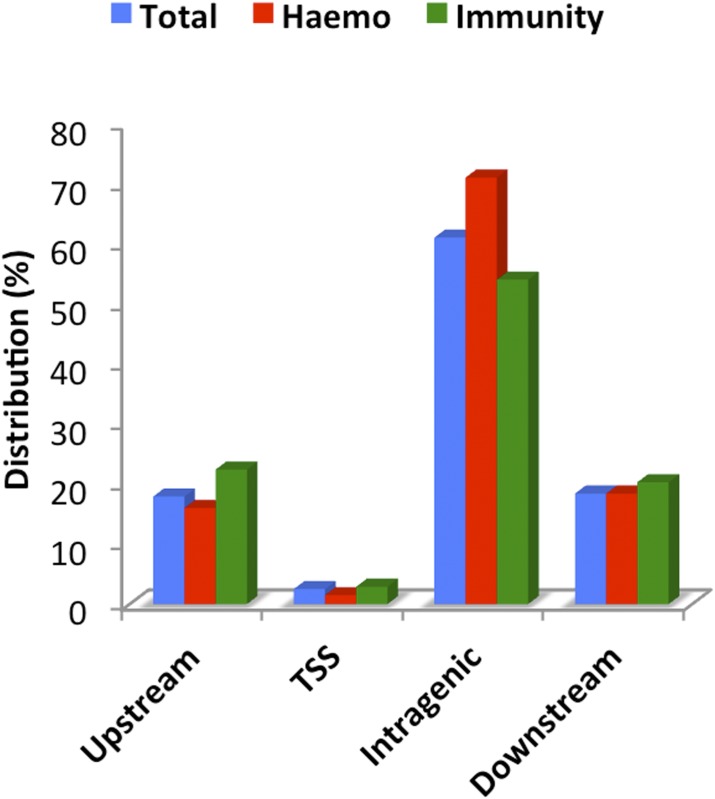
Genomic distribution of *cis*-regulatory sites relative to gene annotations. Genomic distribution of proximal *cis*-regulatory sites relative to gene annotations. The distribution of sites proximal to hematopoietic (Hemo, red) and immunity genes (Immunity, green) is compared to the distribution of sites proximal to all gene annotations (Total, blue). Genomic regions were defined as upstream for up to two kilo bases upstream of the annotated TSS. The TSS fraction comprises from coordinate -60 to coordinate +40 base pairs from the TSS. Intragenic for the region +41 base pairs from the TSS to the 3-prime end. Downstream for the region including up to 2 kilo bases from the 3-prime end of gene annotations.

Next, the genomic distribution of the open chromatin sites identified, relative to the VectorBase gene-annotation data (http://www.vectorbase.org, *A*. *gambiae* PEST, AgamP3), was determined [20, 21]. A proximal upstream fraction was defined as up to 2 Kb upstream of a gene annotation. Similarly, a downstream fraction was defined as up to 2 Kb downstream of a gene annotation. Intragenic sites were those mapping within the 5-prime and 3-prime end annotations of transcripts. Sites were defined as overlapping TSSs when mapping within -60 and +40 bp from the TSS. Finally, sites were defined as distal when mapping outside of all the previously defined regions. Distal open chromatin sites (more than 2 Kb away from any gene annotation) corresponded to 45.22% of the total sites. The remaining 54.78% of the newly identified open chromatin sites mapped within 2 Kb of the gene annotations (Fig 2). This genomic distribution of the proximal sites is distinct from that found in other metazoans, including *Drosophila* [14] and humans [22, 23]. However, in the case of these two species, various cell types and developmental stages have been considered, in contrast with our study of a single cell type. Nevertheless, what *A*. *gambiae* shares with those two species is the largest single genomic fraction of 4a-3B *cis*-regulatory sites, which is the distal. In order to compare total, hematopoietic and immunity gene sites, the sites proximal to gene annotations [24, 25] were considered. The genomic distribution of these proximal sites is depicted as blue bars in Fig 2. Taken together, the reproducibility of detection and the genomic distribution of open chromatin sites indicates an appropriate genome-wide identification of *cis*-regulatory sites.

According to the phenotype of this hemocyte-like immune-responsive cell line, it was expected that some *cis*-regulatory sites, proximal to hematopoietic and immunity genes, would appear in an open chromatin conformation. First, *A*. *gambiae* orthologous genes to *Drosophila* hematopoietic and immunity genes [26] were identified in public databases. A search for *cis*-regulatory sites mapping within 2 Kb of either end of *A*. *gambiae* hematopoietic and immunity gene annotations was carried out. More than one hundred open chromatin *cis*-regulatory sites were shown to map within two Kb of the hematopoietic gene annotations. The genomic distribution of this subset of *cis*-regulatory sites is included in Fig 2 as red bars. A similar search produced more than two hundred *cis*-regulatory sites mapping within two Kb of immunity gene annotations. The distribution of these immunity gene *cis*-regulatory sites is shown in Fig 2, as green bars. The comparison of total, hematopoietic and immunity sites shows that they have differential enrichment of genomic fractions. Immunity gene annotations are enriched for proximal upstream (22.4%) and TSS-overlapping (2.9%) *cis*-regulatory sites, in comparison to hematopoietic and total *cis*-regulatory sites. This suggests that the promoters of some immunity genes are already in an open chromatin conformation, active or poised for transcription. These findings are consistent with the immune-responsive phenotype of 4a-3B cells. Together, proximal hematopoietic *cis*-regulatory sites and proximal immunity *cis*-regulatory sites constitute 3.5% of the total sites in 4a-3B cells. This could constitute an underestimation of sites resulting from the high statistical astringency used for their detection (*P* < 1 x 10^−8^). In addition, a relatively short distance (2Kb from either end of the gene annotations) was applied when selecting the proximal sites. These parameters might seem too astringent, but it is important to note that there are no previous *A*. *gambiae cis*-regulatory site datasets with which to compare them. Furthermore, there are no significant numbers of previously validated *cis*-regulatory sites that could be used as positive controls. Therefore, here we are referring to the sites identified with the highest confidence, although there may be others (general, proximal to hematopoietic genes and proximal to immunity-gene sites).

### Immunity-related motifs are overrepresented in *cis*-regulatory sites

To identify *cis*-regulatory sites and elements, potentially binding immunity-related transcription factors (TFs), a *de novo* motif discovery was carried out with MEME-ChIP from the Suite motif analysis tools [27, 28]. The discovered motifs were compared with *Drosophila*-derived transcription factor binding sites (TFBS) by means of TOMTOM [29]. Many of the sequence motifs identified in our dataset are similar to the *Drosophila* TFBSs. Consistent with the hemocyte-like phenotype of the 4a-3B cells, six sequence motifs similar to the *Drosophila* hematopoietic TF *serpent* binding site were identified (top *srp* site *E* 1.2 x 10^−14^). Furthermore, many sequence motifs similar to the binding sites of immunity TFs were also identified within this dataset. The top enriched motif (*E* 5.2x10^-155^), which is similar to a known TFBS, matches the binding site of *Drosophila* immunity lola-type TF. This TF participates in the antimicrobial humoral response [30], as well as in the larval lymph-gland hematopoiesis of *Drosophila* [31]. An *A*. *gambiae* putative orthologous gene to the *Drosophila lola* gene (FBgn0005630; FBgn0283521) has been identified: It is gene AGAP005245, located on chromosome 2L, as recorded in ensembl! [32] and Genomicus V3.1 [33]. Another highly enriched motif (*E* 7.4 x 10^−17^) of interest is similar to the binding site of epidermal and immunity *Drosophila* Deformed epidermal auto-regulatory factor (Deaf1). This factor has recently been shown to participate in the innate immune response of *Drosophila* [34, 35]. Therefore, our finding that a motif similar to the TFBS for this factor is overrepresented in the set of *cis*-regulatory sites suggests that this TF could be playing a similar role in the innate immunity process of *A*. *gambiae*. The gene with ID AGAP004905 in *A*. *gambiae* has been identified as the orthologue to *Drosophila*’s Deaf1. There were also additional enriched motifs similar to Dorsal-type TFBS (*E* 1.4 x 10^−20^), to CEBP-type TFBSs (*E* 1.6 x 10^−14^), and to lola-type TFBS (*E* 8.9 x10^-26^).

### Identification of immunity-related *cis*-regulatory modules

**Fig 3:**
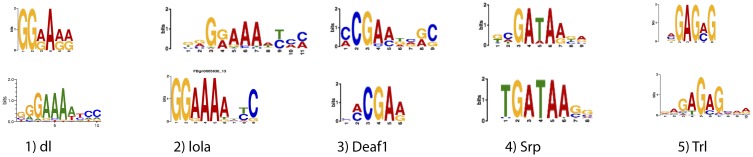
Identified *cis*-regulatory sites contain motifs relevant for gene regulation in immunity. Highly significant motifs identified by *de novo* motif discovery (MEME-ChIP) in the set of *cis*-regulatory sequences. Discovered motifs matching immunity-related TFBSs with an *E* < 1 x 10^−4^. Top logos represent motifs found in *A*. *gambiae cis*-regulatory sequences identified in this work. Bottom logos represent similar *Drosophila* motifs from public databases. The name of the *Drosophila* transcription factor binding each motif is also indicated.

Transcription factors often form multiprotein complexes on top of *cis*-regulatory sequences to control the expression of their target genes. A *cis*-regulatory module (CRM) is a cluster of transcription factor-binding sites that coordinates the cooperative interaction of TFs, and it is often distal to the TSS of the gene it regulates. To investigate the potential cooperation between immunity-related transcription factors and to identify the immunity-related *A*. *gambiae cis*-regulatory modules (CRMs), the co-occurrence of motifs representing TFBSs was investigated. In order to identify the CRMs, the analysis of the *cis*-regulatory sequences identified in this work was carried out by SIOMICS, an algorithm capable of detecting co-occurring motifs within heterogeneous samples [36]. One of the classes of CRMs identified here includes three distinct motifs, and it is similar to Dorsal TFBS (dl-A *E* 2.9 x10^-4^; dl-A *E* 7.9x10^-8^; dl-A *E* 9.9x10^-4^). These three motifs co-occur in 46 *cis*-regulatory sequences. This finding and the frequency of this type of CRM show that it is a key feature of many immunity-related *cis*-regulatory sequences. This is consistent with the clustering of TFBSs in the case of REL-type TFs and with the cooperative interactions previously described for the promoter region of the *A*. *gambiae Def1* gene [6]. Sixty six open chromatin sites were found to contain at least one such motif and map within 5 Kb upstream of an immunity gene (S1 File: **Dataset**). This is consistent with the relevance of the REL-type TFs involved in the mosquitoes’ innate immunity. A motif similar to the STAT-type TFBS co-occurs in a CRM with a motif similar to the REL-type TFBS. This STAT-type motif also co-occurs with the Deaf1-e and dl-type motifs. Members of the JAK/STAT immunity pathway have been identified in *A*. *gambiae*, including two STAT transcription factors: STAT–A and STAT-B [8, 37]. A motif similar to the TFBSs for members of the RUNT domain family of TFs was also found to co-occur with motifs similar to the REL-type TFBSs. In *Aedes aegypti*, the RUNT-related TF 4 (RUNX4) has been shown to cooperate with REL1 in the control of the pro-phenol-oxidase gene expression [38]. Our data suggest that this CRM could also participate in the control of the gene expression during *A*. *gambiae*’s immune response, which also points to the cooperation between these two types of TFs. A motif similar to the Deaf1-type TFBS in *Drosophila* appears in a novel CRM, together with motifs similar to immunity REL-type TFBSs. A highly significant motif (*E val* 5.8 x 10^−59^), similar to a Trl (Trithorax)-like binding site, co-occurs in 52 *cis*-regualtory sequences with a lola-type top motif. Trl is a GAGA-type transcription factor that has been shown to counteract the effects of chromatin repression in *Drosophila*, maintaining open chromatin and recruiting RNA Pol II [39]. The *cis*-regulatory module identified here suggests that the mosquitoes’ Trl factor could maintain open chromatin at *cis*-regulatory sites, where immunity TFs can bind. The example motifs described in the Results section are summarized in Fig 3.

## Discussion and conclusion

Innate immunity effector functions are directly coded in the genome. Therefore, *cis*-regulatory sequences are key components of the gene regulatory networks involved in the innate immune response. In our study, open chromatin profiling was applied to identify *cis*-regulatory sequences potentially involved in the genetic control of *A*. *gambiae*’s innate immune response. The *cis*-regulatory sequences identified in this work include sites mapping to proximal regions of immunity and hematopoietic gene annotations. These findings are consistent with the immune-responsive phenotype of hemocyte-like *A*. *gambiae*-derived model cell line 4a-3B. The sequence analysis using a *de novo* motif discovery revealed a heterogeneous array of motifs. This was expected, given that FAIRE is able to detect all classes of *cis*-regulatory sites as long as they are in an open chromatin conformation. Nevertheless, among the highly significant motifs identified, many were similar to immunity-related TFBSs.

As a reference point, we focused on motifs similar to the REL-type TFBSs, since this type of TFs have been shown to regulate the expression of innate immunity genes in *A*. *gambiae*, *in vivo* and *in vitro*. Therefore, we investigated which other motifs co-occur with this type. Focusing on SIOMICS-detected CRMs containing a motif with the better matching STAMP *E value* (9.2 x10^-8^) to the Dorsal (dl)-type TFBS, the following REL potential partners were identified: REL, STAT, lola and Deaf1. These findings suggest that *Ag*Deaf1 and *Ag*lola are robust innate immunity TF candidates, and that their function in innate immunity could be conserved both in *Drosophila* and *A*. *gambiae*. These findings extend the potential set of TFs that may participate in the genetic control of the innate immune response of *A*. *gambiae*. Some of the motifs found in significant modules do not match known motifs in public databases. These constitute newly discovered putative binding sites for TFs yet to be identified. Taken together, the findings on motifs and CRMs suggest an extended TF combinatorial potential of the transcriptional regulation of the innate immunity response in *A*. *gambiae*. A potential caveat of this study could relate to the fact that 4a-3B has been shown to have some cell-lineage heterogeneity [11]. However, the FAIRE assay, combined with a DFilter peak detection, has been shown to detect specific *cis*-regulatory sites in heterogeneous tissue samples [16]. Nevertheless, this work adds newly identified immunity-related *A*.*gambiae cis*-regulatory sequences, motifs and CRMs. In this study, the motifs and modules discovered in *cis*-regulatory sites are robust TFBS and *cis*-regulatory module candidates that predict the participation of their cognate factors in the innate immunity gene regulation of *A*. *gambiae*. The presence of motifs similar to immunity TFBSs predicts multiple protein-DNA and protein-protein interactions potentially involved in the innate immune response of *A*. *gambiae*. The set of *cis*-regulatory sequences identified in this work is a valuable complement to the *A*. *gambiae* immune response transcriptomes previously described [2–4]. These *cis*-regulatory elements will be useful to further define the molecular mechanisms that participate in the genetic control of the innate immune response in *A*. *gambiae*. In conclusion, the data presented in this work uncover the potential participation of Deaf1- and lola-type TFs in *A*. *gambiae*’s innate immunity process, suggesting the potential conservation of the immunity molecular networks in both this mosquito species and *Drosophila*.

## Materials and methods

### Cell culture

The 4a-3B, *Anopheles gambiae*-derived cell line (MRA-919), was cultured in Schneider´s *Drosophila* medium, supplemented with 10% FCS, as previously described [11].

### Open chromatin profiling

Two biological replicates of 10^8^ 4a3B cells, each in culture, were used to prepare FAIRE DNA. Cells were cultured in duplicate and fixed in formaldehyde; then chromatin was extracted. Chromatin was sheared in a Biorruptor UCD200 to obtain fragments (200 to 500 base pairs in length), prior to chemical fractionation. The fractionation of sheared chromatin was carried out by phenol-chloroform extraction in order to obtain DNA samples enriched in nucleosome-depleted regions according to the FAIRE method, as previously described [13]. These samples were then purified and cleaned using Zymo Research clean and concentration columns, following the manufacturer’s instructions. The DNA from each replicate was used to create a library, and both libraries were subsequently sequenced. DNA samples enriched in open chromatin fragments were then assembled into ChIP-type DNA fragment libraries for massive parallel sequencing.

### Sequencing and mapping

Single-end 36 cycle high-throughput massively parallel sequencing of libraries was carried out on an Illumina GA IIx instrument (Instituto de Biotecnología, Universidad Nacional Autónoma de México). Sequence tags were filtered by quality and aligned to the *A*. *gambiae* genome assembly AgamP4 *Anopheles gambiae* PEST, available at VectorBase (http://www.vectorbase.org) [20, 21, 24, 25]. One library produced 7 240 988 q30 unique reads, and the replicate library produced 8 887 301 q30 unique reads. Unique non-duplicated aligned sequence tags were used for open chromatin site peak calling. Prior to peak calling, aligned and filtered sequence tag datasets were validated by CHip-seq ANalytics and Confidence Estimation (CHANCE) software (https://github.com/songlab/chance).

### Open chromatin site peak calling

A set of *cis*-regulatory sites, which could be useful to contrast our results with, is currently lacking. Therefore, the data were analyzed for peak detection using two algorithms with their respective software programs. We used the Model-based Analysis for ChIP-Seq software (MACS) [40] and the DFilter software, both previously validated for FAIRE-seq samples [16]. The set of peaks was obtained by intersecting MACS called peaks with DFilter called peaks (both with a threshold *P* value of 1x10^-8^). For the purpose of the *de novo* motif discovery, a 600-bp sequence length was considered for all detected sites, centered on the peak summit (most enriched base pair for each detected peak). The comparison between the replicates were performed using BEDTools, version 2.17 [41, 42] and MAnorm [17]. FAIRE-seq can detect *cis*-regulatory regions with similar sensitivity to DNaseI-seq, when an increased number of tags are used for peak calling [16]. In this study, we used a number of sequence tags similar to that recommended for this type of data, according to the genome size [43]. To validate the peak-calling process, reproducibility of the *cis*-regulatory sites identified here was carried out on selected sites by FAIRE-qPCR and DNaseI-qPCR verification. Selected sites were confirmed in independently prepared open chromatin samples. Tested sites were chosen as examples of proximal, distal and intragenic *cis*-regulatory sites. The reproducibility of the peaks identified at genome-wide scale between replicates was determined as described in S2 File: Supporting Information (Methods).

### *De novo* motif discovery in *cis*-regulatory sequences and motif comparison

The sequences corresponding to the open chromatin sites identified in this work were masked using the Repeat Masker server [44]. They were subsequently subjected to sequence composition analysis for the *de novo* motif discovery and the detection of *cis*-regulatory modules (CRMs). The motif analysis was carried out with the MEME-ChIP sequence analysis tool [27, 28]. The motif co-occurrence analysis was carried out using the SIOMICS software version V1.4 [36, 45] in order to discover motifs corresponding to potential TFBSs and their predicted interactions. For this analysis, a sequence length of six base-pairs was used as a seed; a *p-*value threshold of 10^−4^ and 20 analysis iterations were also set. The DNA motif comparison within SIOMICS was carried out with the STAMP suite, applying the Pearson Correlation Coefficient for column comparison and the ungapped Smith-Waterman alignment method to find the best matching motifs within the modules. The discovered motifs were compared against curated motif databases, including FlyReg (Bergman/Pollard) (http://www.benoslab.pitt.edu/stamp/) [46, 47].

### Real Time PCR reactions

The oligonucleotides for the FAIRE-qPCR were designed and validated by PCR, melting curve analysis, and qPCR dynamic range. The Real Time PCR reactions were carried out with the SYBR Advantage reagent (Clontech) in a FAST 7500 Applied Biosystems Thermal Cycler. Enrichment was calculated using the comparative Ct method [48] with a control DNA sample as a reference for each site. Enrichment for all sites was normalized with an amplicon directed to a region where no open chromatin sites were detected. The oligonucleotide sequences are listed in Supplemental Methods, Table A in S2 File.

### DNaseI sensitivity assay

The DNaseI-qPCR analysis was carried out as previously described [49]. The oligonucleotides were the same ones used for the FAIRE-qPCR (Table A in S2 File), and the qPCR reactions were carried out as above.

## Supporting information

S1 File Dataset. Genomic coordinates of the summits of 26 440 open chromatin *cis*-regulatory sites identified by FAIRE-seq and overlapping detection by DFilter and MACS.
(XLSX)

S2 File Supplemental methods. Supplemental Methods, supplemental Tables and supplemental Figures.
(PDF)
