# Supplementary material for: Identification of cis-regulatory sequences reveals potential participation of lola and Deaf1 transcription factors in Anopheles gambiae innate immune response
Source: PLoS One. 2017 Oct 13;12(10):e0186435. doi: 10.1371/journal.pone.0186435 (PMC5640250; doi:10.1371/journal.pone.0186435)
Supplement: S2 File — Supplemental Methods, supplemental Tables and supplemental Figures. (PDF) [file pone.0186435.s002.pdf]

Supplemental Methods

**Open chromatin site**	**Forward** (5´to 3´)	**Reverse** (5´to 3´)
AgSrp -10K	AGGAGGAGGAACAACGAA	CTCACACCCTTACACACA
AgSrp -4K	GGAAGCAGGAGAAAGAGAA	CGTGTCAAAGCGAGATCAA
AgSrp +8K	GTGTATGTGGTTCCCTTTT	CACCGTAGTTGCATGATTT
AgPnr -2K	ACCACGCCCTCTTTTTATG	AAATACGCACCACCAACC
AgPnr +7K	TGTTTATGTTAGGCGCAG	TAAAAACAGAACGAACCCG
AgPnr +11K	ACGTTGATGACAGGTTCC	ATCAACGACGAAATCCCG
AgPnr +12K	ACTAATAAGCCACAGACGG	CAAACTCACAACTCACCAAAC
Ag non-open region	GGAGATAGTGTGAGCGTTGTC	TCGATATCCGCTTGCACATT

Table A. Oligonucleotides used to validate by qPCR selected *cis* regulatory sites identified in 4a3B cells.

Pre-processing of fastq sequencing files

Sequence tags were aligned to *Anopheles gambiae* genome using BWA. Aligned sequences were then sorted and selected by quality (q30 threshold) using samtools. Duplicate tags were removed from the resulting files. Files were then converted to the bed format (with BamTools) prior to peak detection with MACS and DFilter.

Determination of reproducible peaks between biological replicates

DFilter is a program that "trains itself" to differentiate between positively and negatively enriched regions from the data used as input. It maximizes the ratio of the mean of difference between true signal and noise to its standard deviation; this can be thought as a z-score. The criterion of finding these regions is based on the idea of moving in windows encompassing a defined number of base pairs (bins). The number of tags inside those bins is counted, the mean and covariance (separataley for positives and negatives) is used in the calculations. In order to define these sets of positive and negative regions DFilter firsts prioritizes regions that have a score greater than 2 standard deviations above the mean, it then merges adjacent regions, discards outliers and the remaining 1,000 are declared positives. After self-training, it computes the values of the coefficients of the detection filter that will be applied to the rest of the genome. For this work we decided to use windows of 40 bins of 5 bp each one (200bp) in order to make focal positive signal findings. We had two replicates. The difference in the number of peaks detected between replicates led us to think that the most straightforward way to keep the reliable information was to only use the best (in terms of p-value, e^{-5}, calculated using the maximum score) peaks shared between replicates.

Replicates were also compared using **MAnorm**.

a)

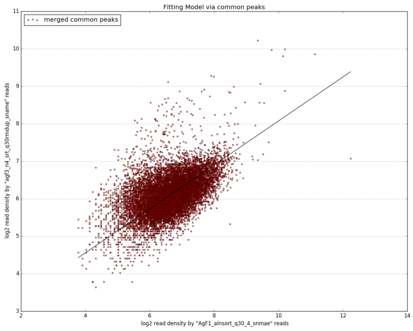

b)

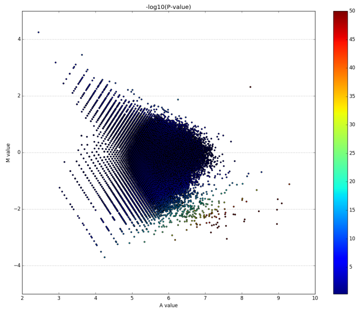

c)

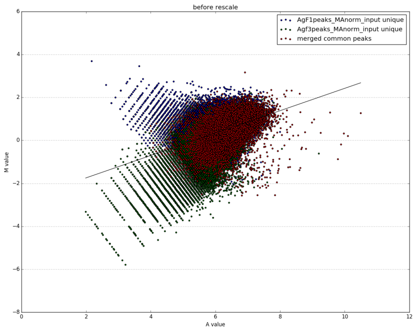

d)

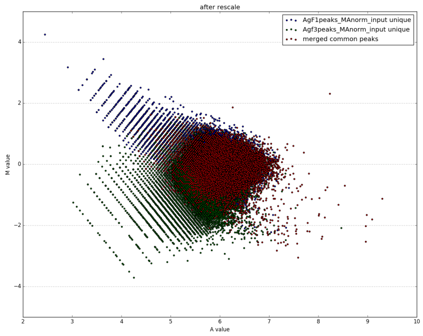

Figure A. Comparison of peaks detected in replicate samples. **a)** Read density comparison. **b)** *P value* comparison. **c)** Peak comparison before rescale. **d)** Peak comparison after rescale.
